# 

**Published:** 1911-03-15

**Authors:** 


CONTENTS

Event and Comment - - - - - - - - - - - - - - - - - - 109
Ante-Natal Physiology, - - - - - - *By N. S. Hoff, D.D.S.* 112
Isn't It Pathetic, - - - - - - - - - *By James W. Cormany.* 120
Prophylaxis, - - - - - - *By Edward C. Kirk, D.D.S., SC.D.* 127
The Use of Boiled Drinking Water, and Calcification in Childhood and Youth, - - - - - - - - - - - - - *By J. Ferrier.* 131
Ethyl Chlorid Anesthesia by the Open Method, *By C. Edson Abbott, D.D.S.* 132
Local Anesthesia in Dentistry, . - - - - *By Guido Fischer.* 134
Bibliography, - - - - - - - - - - - - - - - - - - - - - - 145
Indents - - - - - - - - - - - - - - - - - - - - - - 147
Announcements - - - - - - - - - - - - - - - - - - - - 154

